# The anti-inflammatory function of HDL is impaired in type 2 diabetes: role of hyperglycemia, paraoxonase-1 and low grade inflammation

**DOI:** 10.1186/s12933-017-0613-8

**Published:** 2017-10-12

**Authors:** Sanam Ebtehaj, Eke G. Gruppen, Mojtaba Parvizi, Uwe J. F. Tietge, Robin P. F. Dullaart

**Affiliations:** 1 Department of Pediatrics, University of Groningen, University Medical Center Groningen, Hanzeplein 1, 9713GZ Groningen, The Netherlands; 2 Department of Endocrinology, University of Groningen, University Medical Center Groningen, Hanzeplein 1, 9713GZ Groningen, The Netherlands; 3 Department of Pathology and Medical Biology, University of Groningen, University Medical Center Groningen, Hanzeplein 1, 9713GZ Groningen, The Netherlands

**Keywords:** High sensitivity C-reactive protein, High density lipoprotein function, Paraoxonase-1, Serum amyloid A, Type 2 diabetes mellitus

## Abstract

**Background:**

Functional properties of high density lipoproteins (HDL) are increasingly recognized to play a physiological role in atheroprotection. Type 2 diabetes mellitus (T2DM) is characterized by low HDL cholesterol, but the effect of chronic hyperglycemia on the anti-inflammatory capacity of HDL, a metric of HDL function, is unclear. Therefore, the aim of the present study was to establish the impact of T2DM on the HDL anti-inflammatory capacity, taking paraoxonase-1 (PON-1) activity and low grade inflammation into account.

**Methods:**

The HDL anti-inflammatory capacity, determined as the ability to suppress tumor necrosis factor-α (TNF-α) induced vascular cell adhesion molecule-1 (VCAM-1) mRNA expression in endothelial cells in vitro (higher values indicate lower anti-inflammatory capacity), PON-1 (arylesterase) activity, hs-C-reactive protein (hs-CRP), serum amyloid A (SAA) and TNF-α were compared in 40 subjects with T2DM (no insulin or statin treatment) and 36 non-diabetic subjects.

**Results:**

T2DM was associated with impaired HDL anti-inflammatory capacity (3.18 vs 1.05 fold increase in VCAM-1 mRNA expression; *P* < 0.001), coinciding with decreased HDL cholesterol (*P* = 0.001), apolipoprotein A-I (*P* = 0.038) and PON-1 activity (*P* = 0.023), as well as increased hs-CRP (*P* = 0.043) and TNF-α (*P* = 0.005). In all subjects combined, age- and sex-adjusted multivariable linear regression analysis demonstrated that impaired HDL anti-inflammatory capacity was associated with hyperglycemia (β = 0.499, *P* < 0.001), lower PON-1 activity (β = − 0.192, *P* = 0.030) and higher hs-CRP (β = 0.220, *P* = 0.016).

**Conclusions:**

The HDL anti-inflammatory capacity is substantially impaired in T2DM, at least partly attributable to the degree of hyperglycemia, decreased PON-1 activity and enhanced low grade chronic inflammation. Decreased anti-inflammatory protection capacity of HDL conceivably contributes to the increased atherosclerosis risk associated with T2DM.

## Introduction

Patients with type 2 diabetes mellitus (T2DM) have a substantially increased risk of atherosclerotic cardiovascular disease (CVD) [1]. This elevated CVD risk has been, at least in part, attributed to the low high density lipoprotein (HDL) cholesterol levels consistently found in patients with insulin resistance and T2DM [2–4]. However, data from genetic as well as pharmacological intervention studies now suggest that HDL cholesterol levels per se might not be as relevant as a predictive biomarker of atherosclerosis as previously thought [5–7]. Instead, the functionality of HDL particles could have a more pathophysiologically important impact [8, 9], together with determinations of HDL subpopulations and HDL particle numbers [10–14]. HDL may slow the process of atherogenesis by several important biological properties. First, HDL promotes cholesterol efflux and reverse cholesterol transport, thereby removing cholesterol from macrophage foam cells in atherosclerotic lesions [8, 9]. Variable results have been reported on the impact of diabetes on HDL-mediated cholesterol efflux capacity, likely due to different assay conditions employed and reflecting the influence of the severity of hyperglycemia [15–17]. In a recent cross-sectional analysis of the CODAM study including 552 subjects we did not find evidence that moderate hyperglycemia as such impairs cholesterol efflux capacity [17]. Second, HDL can inhibit the oxidation of native LDL thereby preventing the formation of pro-atherosclerotic and pro-inflammatory LDL particles [18]. Also for the effect of T2DM on the anti-oxidative capacity of HDL opposing data have been reported. In some studies, HDL from T2DM patients exhibited a reduced capacity to protect low density lipoproteins (LDL) from oxidation [19–21], whereas we found previously that the anti-oxidative capacity of HDL is impaired in T2DM but only taking account of the diabetes-associated decrease in HDL cholesterol [22]. Again, these differences might be related to the degree of metabolic control in the T2DM patients studied. Third, HDL has potent anti-inflammatory activity and can reduce, e.g. endothelial activation [8, 9, 23], thus also interfering with an early step of atherogenesis. Notably, the anti-inflammatory capacity of HDL was found to be impaired in acute myocardial infarction patients [24–26], and to predict recurrent CVD events even independent of HDL cholesterol and apolipoprotein (apo) A-I, the class-defining apolipoprotein of HDL [25]. Much less is currently known about the potential effect of T2DM specifically on the anti-inflammatory properties of HDL.

In the present cross-sectional study we tested the hypothesis that the anti-inflammatory function of HDL is impaired in T2DM. Furthermore, we aimed to delineate the relationship of this metric of HDL function with hyperglycemia, PON-1 activity and low grade chronic inflammation markers.

## Methods

### Subjects

The study protocol was approved by the medical ethics committee of the University Medical Center Groningen. Men and women with and without T2DM, aged > 18 years, were recruited by advertisement in local newspapers. They participated after written informed consent had been obtained. T2DM had been diagnosed previously according to guidelines from the Dutch College of General Practitioners (fasting plasma glucose ≥ 7.0 mmol/L; non-fasting plasma glucose ≥ 11.1 mmol/L). Subjects with a history of CVD, chronic kidney disease (estimated glomerular filtration rate < 60 mL/min/1.73 m^2^ and/or proteinuria), liver function abnormalities or thyroid dysfunction, as well as current smokers and subjects who used lipid lowering drugs were also excluded. The use of metformin, sulfonylurea and antihypertensive medication was allowed, but insulin use was an exclusion criterion. Blood pressure was measured after 15 min rest at the left arm in sitting position using a sphygmomanometer. Body mass index (BMI in kg/m^2^) was calculated as weight divided by height squared. Waist circumference was measured between the 10th rib and the iliac crest. The metabolic syndrome (MetS) was defined according to the revised NCEP-ATP III criteria [27]. Three or more of the following criteria were required for categorization of subjects with MetS: waist circumference > 102 cm for men and > 88 cm for women, hypertension (blood pressure ≥ 130/85 mmHg or use of antihypertensive medication), fasting plasma triglycerides ≥ 1.70 mmol/L; HDL cholesterol < 1.0 mmol/L for men and < 1.3 mmol/L for women, fasting glucose ≥ 5.6 mmol/L, or previously diagnosed T2DM. The participants were studied after an overnight fast.

### Laboratory analyses

Serum and EDTA-anticoagulated plasma samples were stored at − 80 °C until analysis. Plasma glucose was measured shortly after blood collection with an APEC glucose analyzer (APEC Inc., Danvers, MA, USA).

Total cholesterol and triglycerides were measured by routine enzymatic methods (Roche/Hitachi Cat. Nos 11875540 and 1187602, respectively; Roche Diagnostics GmBH, Mannheim, Germany). HDL cholesterol was assayed by a homogeneous enzymatic colorimetric test (Roche/Hitachi, Cat. No 04713214). Non-HDL cholesterol was calculated as the difference between total cholesterol and HDL cholesterol. LDL cholesterol was calculated by the Friedewald formula if triglycerides were < 4.5 mmol/L. Apolipoprotein A-I (apoA-I) and apolipoprotein B (apoB) were measured by immunoturbidimetry (Roche/Cobas Integra Tinaquant Cat No. 03032566, Roche Diagnostics). Glycated hemoglobin (HbA1c) was measured by high-performance liquid chromatography (Bio-Rad, Veenendaal, the Netherlands; normal range: 27–43 mmol/mol).

Serum paraoxonase-1 (PON-1) enzymatic activity was measured as arylesterase activity, i.e. as the rate of hydrolysis of phenyl acetate into phenol [28, 29]. Arylesterase activity, measured with this assay, is positively correlated with PON-1 enzymatic activity toward paraoxon [30].

High sensitivity C-reactive protein (hs-CRP) was assayed by nephelometry with a lower limit of 0.175 mg/L (BNII N; Dade Behring, Marburg, Germany). The serum amyloid A (SAA) protein was assayed by a monoclonal antibody-based sandwich SAA1 enzyme-linked immunosorbent assay (ELISA) [31, 32]. Human apo-SAA was purified from the HDL3 fraction of acute phase serum, linked to helix pomatia haemocyanin, and subsequently injected into Balb/c mice to produce monoclonal antihuman-SAA antibodies. The antibodies used in the sandwich ELISA are the capture antibody Reu.86.5, which reacts to all acute phase SAA subtypes, and the coupled to Horseradish peroxidase detection antibody Reu.86.1, which reacts with the major SAA1 subtype. The assay is standardized against the international standard for SAA protein (WHO code 92/680). The lower limit of detection of the assay is 1.6 µg/L. Plasma TNF-α was measured using Luminex xMAP technology (Lincoplex panel B Cat. No. HADK1-61K-B; Linco Research Inc., St. Charles, MO, USA) [33]. TNF-α levels, measured with this technology, are strongly correlated (r > 0.80) with assay results obtained by enzyme-linked immunoassays obtained from Linco Inc. (data provided by the manufacturer). The intra-assay coefficients of variation (CV) of all these assays are ≤ 8.0%.

The HDL anti-inflammatory capacity was determined using an in vitro cell system essentially following a recently described procedure [25, 26]. ApoB-containing lipoproteins were precipitated from plasma by adding 100 μL 36% polyethylene glycol (PEG 6000, Sigma, St. Louis, MO, USA) in 10 mM HEPES (pH = 8.0) to 200 μL plasma followed by 30 min incubation on ice. After 30 min centrifugation at 2200*g*, the HDL-containing supernatant was collected, kept on ice, and used directly for the HDL anti-inflammatory assay. Human umbilical vein endothelial cells (HUVECs), pooled from at least 8 different donors, were provided by the Endothelial Cell Core Facility of the University of Groningen, The Netherlands, and pre-incubated for 30 min with 2% of individual apoB-depleted plasma samples or with equal amounts of phosphate buffered saline (PBS) as control [34]. Subsequently, 10 ng/mL TNF-α (R&D systems, Abingdon, UK) or PBS as control was added, and cells were incubated for an additional 5 h followed by analysis of vascular cell adhesion molecule-1 (VCAM-1) gene expression using quantitative real-time polymerase chain reaction. VCAM-1 mRNA expression was calculated relative to the average of the housekeeping gene cyclophilin [34]. Results were further normalized to the average of the relative VCAM-1 expression of the control group. Individual values represent fold induction over control values, whereby higher values indicate less suppression of VCAM-1 induction, i.e. lower anti-inflammatory capacity. In addition, under the assay conditions employed neither control HDL nor HDL from severely hyperglycemic patients affects the viability of the HUVECs (data not shown). Storage of plasma at − 80 °C did not influence the results of this assay [26]. The intra-assay CV of this assay is 9% [26]. To limit potential variation due to different assay conditions, measurements of the anti-inflammatory function of HDL were carried out at the same time using the same batch of pooled cells and the same reagents.

### Statistical analysis

IBM SPSS software (SPSS, version 22.0, SPSS Inc. Chicago, IL, USA) was used for data analysis. Results are expressed as mean ± SD or as median (interquartile range). Because of skewed distribution, natural logarithm (log_e_) transformed values of HDL anti-inflammatory capacity, triglycerides, hs-CRP, SAA and TNF-α were used. Between group differences in variables were determined by unpaired t tests or by Chi square tests. Univariate correlations were determined by Pearson correlation coefficients. Multivariable linear regression analyses were carried out to disclose the independent relationships of HDL anti-inflammatory capacity with diabetes status, MetS classification, PON-1 activity and inflammation markers. Two-sided *P* < 0.05 were considered significant.

## Results

Forty diabetic subjects (19 men/21 women) and 36 non-diabetic subjects (8 men/28 women) were included in the study (Table 1). Twenty-eight T2DM subjects and 7 non-diabetic subjects were classified with MetS (*P* < 0.001). Among diabetic participants, diabetes duration was 5.0 (4.0–6.7) years. All diabetic subjects had received diet advice as part of their routine medical care. Eight T2DM subjects were taking metformin alone and seven were taking sulfonylurea alone. Both drugs were used by 13 T2DM participants. Other glucose lowering drugs were not taken. Twelve diabetic participants did not use glucose lowering drugs. Anti-hypertensive medication (mainly angiotensin-converting enzyme inhibitors, angiotensin II receptor antagonists and diuretics, alone or in combination) were used by 18 T2DM subjects. None of the non-diabetic subjects used anti-hypertensive drugs (*P* < 0.001). Three women were using estrogens. Other medications were not taken.

**Table 1:** Clinical characteristics, plasma (apo)lipoproteins, paraoxonase-1 activity, inflammation markers and anti-inflammatory capacity of high density lipoproteins in 40 patients with type 2 diabetes mellitus (T2DM) and in 36 non-diabetic subjects, and univariate correlation coefficients with the HDL anti-inflammatory capacity in all subjects combined

	T2DM subjects (n = 40)	Non-diabetic subjects (n = 36)	*P* value*	Correlation coefficient with HDL anti-inflammatory capacity	*P* value**
Age (years)	60 ± 10	52 ± 9	< 0.001	0.008	0.94
Sex (men/women)	19/21	8/28	0.039		
Systolic blood pressure (mmHg)	145 ± 20	129 ± 20	0.001	0.253	0.028
Diastolic blood pressure (mmHg)	87 ± 9	81 ± 12	0.039	0.184	0.054
BMI (kg/m^2^)	28.9 ± 4.9	25.2 ± 4.1	0.001	0.302	0.008
Waist (cm)	99 ± 14	83 ± 13		0.375	0.001
Glucose (mmol/L)	8.8 ± 2.3	5.6 ± 0.7	< 0.001	0.614	< 0.001
HbA1c (mmol/mol)	50 ± 8	34 ± 3	< 0.001	0.432	< 0.001
Total cholesterol (mmol/L)	5.54 ± 0.97	5.58 ± 0.89	0.87	− 0.024	0.84
Non-HDL cholesterol (mmol/L)	4.24 ± 1.05	3.98 ± 1.00	0.27	0.072	0.54
LDL cholesterol (mmol/L)	3.41 ± 0.88	3.38 ± 0.88	0.91	− 0.024	0.84
HDL cholesterol (mmol/L)	1.30 ± 0.39	1.60 ± 0.35	0.001	− 0.242	0.035
Triglycerides (mmol/L)	1.67 (1.22–2.16)	1.15 (0.88–1.75)	0.004	0.258	0.025
ApoB (g/L)	0.97 ± 0.24	0.88 ± 0.22	0.095	0.120	0.30
ApoA-I (g/L)	1.36 ± 0.26	1.48 ± 0.20	0.038	− 0.140	0.22
PON-1 activity (U/L)	111.2 ± 38.1	131.2 ± 36.3	0.023	− 0.291	0.011
hs-CRP (mg/L)	1.58 (0.99–2.85)	0.94 (0.42–2.47)	0.043	0.253	0.029
SAA (mg/L)	1.71 (1.25–2.48)	1.58 (0.82–2.18)	0.25	0.211	0.067
TNF-α (ng/L)	3.50 (2.80–5.20)	2.95 (2.40–3.48)	0.005	0.240	0.038
HDL anti-inflammatory capacity (fold increase in VCAM-1 mRNA expression)	3.18 (2.17–4.33)	1.05 (0.63–1.38)	< 0.001		

Data are mean ± SD or medians (interquartile range). BMI, body mass index; HbA1c, glycated hemoglobin; apo, apolipoprotein; HDL, high density lipoproteins; apo, apolipoprotein; hs-CRP, high sensitivity C-reactive protein; LDL, low density lipoproteins; PON-1, paraoxonase-1; SAA, serum amyloid A; TNF-α, tumor necrosis factor-α; VCAM-1, vascular cell adhesion molecule-1. LDL cholesterol was calculated in 38 T2DM subjects and in 35 non-diabetic subjects. Pearson correlation coefficients are shown with log_e_ transformed values of triglycerides, hs-CRP, SAA, TNF-α and HDL anti-inflammatory capacity (higher values indicate lower anti-inflammatory capacity). *P* value*: *P* value for the difference between diabetic and non-diabetic subjects; *P* value**: *P* value for the correlation coefficient

The T2DM subjects were older, and more likely to be men than the non-diabetic participants (Table 1). Systolic and diastolic blood pressure, BMI, waist circumference, glucose and HbA1c levels were higher in T2DM subjects. Total cholesterol, non-HDL cholesterol, LDL cholesterol and apoB levels were not different between the groups. Triglyceride levels were higher, whereas HDL cholesterol and apoA-I were lower in T2DM subjects. The HDL anti-inflammatory capacity was strongly impaired in T2DM subjects [3.18 vs 1.05 fold increase in VCAM-1 mRNA expression (higher values indicate lower anti-inflammatory capacity); *P* < 0.001, Table 1], coinciding with lower PON-1 activity, higher hsCRP and TNF-α levels. The difference in SAA between the groups was not significant. Additionally, the HDL anti-inflammatory capacity was worse in all subjects with vs all subjects without MetS combined [3.16 (1.74–4.34) vs 1.24 (0.67–1.96) fold increase in VCAM1 expression, *P* < 0.001]. The HDL anti-inflammatory capacity was also different in the diabetic subjects (n = 40) vs non-diabetic subjects without MetS [n = 29; 1.03 (0.49–1.37) fold increase in VCAM1 expression, *P* < 0.001], but was not different between non-diabetic subjects with and without MetS (*P* = 0.11). Among diabetic subjects, the HDL anti-inflammatory capacity did not significantly differ between men and women [men 3.61 (2.23–4.39) and women 3.02 (2.09–4.18) fold increase in VCAM1 expression; *P* = 0.64]. In non-diabetic subjects, the HDL anti-inflammatory capacity was also not different between men and women [men 0.88 (0.44–1.37) and women: 1.06 (0.63–1.38) fold increase in VCAM1 expression; *P* = 0.64].

**Fig. 1:**
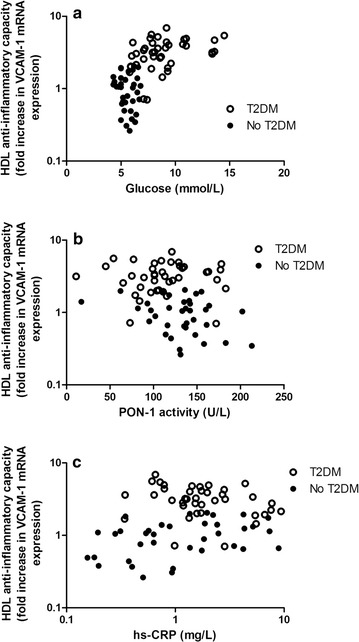
Scatterplots showing univariate relationships of high density lipoprotein (HDL) anti-inflammatory capacity (fold increase in VCAM-1 expression) in 40 subjects with type 2 diabetes mellitus (T2DM) (empty circles) and in 36 subjects without diabetes (filled circles) with plasma glucose (**a**), paraoxonase-1 (PON-1) activity (**b**) and high sensitivity C-reactive protein (hsCRP) (**c**). Higher HDL anti-inflammatory capacity values represent lower HDL anti-inflammatory function. The HDL anti-inflammatory capacity and hsCRP values are log_e_ transformed. Correlation coefficients: **a** all subjects combined: r = 0.614, *P* < 0.001; T2DM subjects; r = 0.357, *P* = 0.024; non-diabetic subjects: r = − 0.127, *P* = 0.46. **b** All subjects combined: r = − 0.291, *P* = 0.011; T2DM subjects; r = 0.014, *P* = 0.93; non-diabetic subjects: r = − 0.315, *P* = 0.061. **c** All subjects combined: r = 0.253, *P* = 0.029; T2DM subjects; r = − 0.258, *P* = 0.11; non-diabetic subjects: r = 0.373, *P* = 0.025

In all subjects combined, the HDL anti-inflammatory capacity values were strongly correlated with glucose and HbA1c (Table 1). HDL anti-inflammatory capacity values were also positively correlated with age, systolic blood pressure, waist circumference, triglycerides, hsCRP and TNF-α, whereas inverse correlations were observed with HDL cholesterol and PON-1 activity (Table 1). In T2DM subjects separately, the HDL anti-inflammatory capacity was correlated with glucose (r = 0.357, *P* = 0.024), whereas in non-diabetic subjects the HDL anti-inflammatory capacity was correlated with hs-CRP (r = 0.373, *P* = 0.025). Figure 1 shows the univariate relationships of the HDL anti-inflammatory capacity with glucose, PON-1 activity and hs-CRP in all subjects combined and in T2DM and non-diabetic subjects separately.

Multivariable linear regression analyses were first carried out to determine whether the HDL anti-inflammatory capacity remained associated with the presence of T2DM when taking account of age, sex, categorization of MetS, and the use of glucose lowering drugs and anti-hypertensive medication. In age- and sex-adjusted analysis, the HDL anti-inflammatory capacity was associated with the presence of T2DM (β = 0.660, *P* < 0.001) independent of MetS categorization (β = 0.148, *P* = 0.10; data not shown). The association of the HDL anti-inflammatory capacity with T2DM was unaltered after additional adjustment for glucose lowering medication and anti-hypertensive drugs (β = 0.705, *P* < 0.001); in this analysis the association of HDL anti-inflammatory capacity with MetS was again not significant (β = 0.155, *P* = 0.11). Given that the HDL anti-inflammatory capacity was also correlated positively with systolic blood pressure, waist circumference and plasma triglycerides, and inversely with HDL cholesterol in all subjects combined (Table 1), we performed further multivariable regression analysis with the HDL anti-inflammatory capacity as dependent variable and these individual MetS-related variables as potential contributing covariates. In such age- and sex-adjusted analyses, the HDL anti-inflammatory capacity was again positively related to the presence of diabetes (β = 0.765, *P* < 0.001) without independent contributions of systolic blood pressure (β = − 0.039, *P* = 0.63), waist circumference (β = − 0.019, *P* = 0.87), triglyceride levels (β = − 0.039, *P* = 0.63) and HDL cholesterol (β = 0.080, *P* = 0.50).

We next evaluated the relationship of the HDL anti-inflammatory capacity with glycemia, PON-1 activity and inflammation markers, representing variables with which the HDL anti-inflammatory capacity was correlated in univariate correlation analysis (Table 1). In age- and sex-adjusted analysis the HDL anti-inflammatory capacity was associated positively with glucose and inversely with PON-1 activity (Table 2A); these relationships were not modified by HDL cholesterol or apoA-I (β = − 0.008; *P* = 0.94 and β = − 0.019; *P* = 0.85, respectively). Of the three inflammation markers, the strongest univariate association with the HDL anti-inflammatory capacity was found for hs-CRP. In agreement, in age- and sex-adjusted analyses the HDL anti-inflammatory capacity was associated with hs-CRP (β = 0.279, *P* = 0.025) but not independently with TNF-α (β = 0.081, *P* = 0.47) and SAA (β = 0.078, *P* = 0.52). hs-CRP was, therefore, chosen for further analysis. In a subsequent model, the HDL anti-inflammatory capacity was positively associated with hs-CRP independent of plasma glucose (Table 2A, Model 2). When glucose, PON-1 activity and hs-CRP were included together in the analysis, the HDL anti-inflammatory capacity was still positively associated with glucose and hs-CRP and inversely with PON-1 activity (Table 2A, Model 3). These relationships were essentially unaltered taking account of glucose lowering drugs and antihypertensive medication (data not shown). In alternative analyses with HbA1c instead of plasma glucose, the HDL anti-inflammatory capacity was positively associated with HbA1c and inversely with PON-1 activity (Table 2B, Model 1), positively with HbA1c and hs-CRP (Table 2B, Model 2), positively with HbA1c and hs-CRP, and inversely with PON-1 activity (Table 2B, Model 3). Combined, these results indicate that the HDL anti-inflammatory capacity is impaired in the context of chronic hyperglycemia, even independent of HDL cholesterol, diabetes-associated impaired PON-1 activity and enhanced low grade chronic inflammation.

**Table 2:** Multivariable linear regression analysis showing associations of the HDL anti-inflammatory capacity (determined as fold increase in VCAM-1 expression) with glucose, glycated hemoglobin, paraoxonase-1 activity and inflammation markers

	Model 1		Model 2		Model 3	
	β	*P* value	β	*P* value	β	*P* value
A						
Age	− 0.118	0.23	0.155	0.120	0.168	0.084
Sex (men/women)	0.046	0.61	0.103	0.26	0.086	0.335
Glucose	0.535	< 0.001	0.521	< 0.001	0.499	< 0.001
PON-1 activity	− 0.240	0.009			− 0.192	0.030
hs-CRP			0.232	0.013	0.220	0.016
B						
Age	0.197	0.059	0.241	0.028	0.253	0.018
Sex (men/women)	0.049	0.63	0.112	0.28	0.093	0.36
HbA1c	0.395	< 0.001	0.345	0.003	0.326	0.004
PON-1 activity	− 0.255	0.010			− 0.216	0.028
hs-CRP			0.223	0.038	0.211	0.044

A: Model 1: includes age, sex, glucose and PON-1 activity as independent variables. Model 2: includes age, sex, glucose and hs-CRP as independent variables. Model 3: includes age, sex, glucose, PON-1 activity and hs-CRP as independent variables

B: Model 1: includes age, sex, HbA1c and PON-1 activity as independent variables. Model 2: includes age, sex, HbA1c and hs-CRP as independent variables. Model 3: includes age, sex, HbA1c, PON-1 activity and hs-CRP as independent variables

β, standardized regression coefficient; PON-1, paraoxonase-1; HbA1c, glycated hemoglobin; hs-CRP, high sensitivity C-reactive protein; the HDL anti-inflammatory capacity and hs-CRP are log_e_ transformed. A positive association indicates a relationship with lower HDL anti-inflammatory capacity

## Discussion

The results of this study demonstrate that the anti-inflammatory properties of HDL are remarkably impaired in T2DM, even in patients with generally acceptable metabolic control. It seems conceivable that such an impaired functionality of HDL may contribute to the increase in risk of atherosclerotic CVD associated with T2DM.

In this study, we used an assay that determines the effect of HDL preparations on the expression of VCAM-1 on endothelial cells [25, 26]. Thereby, the read-out of HDL function is envisaged to directly reflect a critical early event in the process of atherogenesis, namely the recruitment of macrophages into developing atherosclerotic lesions. In this respect, our study adds to previous work showing that HDL from T2DM patients exhibits decreased endothelial cell-dependent vasoprotective properties including reduced NO production, endothelium-dependent vasodilation and reendothelialization after injury [35]. Another study using a different type of assay also reached the conclusion that the diabetic state impacts negatively on the anti-inflammatory function of HDL [21]. This report demonstrated impaired anti-inflammatory activity of HDL in T2DM determined as HDL-mediated inhibition of LDL-induced chemotaxis of macrophages towards endothelial cell-conditioned medium. As a mechanism, the enrichment of HDL with SAA was suggested to explain decreased anti-inflammatory properties of HDL [21]; T2DM may result in increased circulating SAA levels already in young patients [36]. These data are also consistent with previous work in patients with end-stage renal disease [37], in which SAA was indeed identified as underlying factor for impaired HDL anti-inflammatory function. Literature points to a contribution of SAA, which is largely contained within the HDL fraction [38], to attenuated HDL function [8, 37]. However, in our present study we found no significant association of SAA with a decreased HDL anti-inflammatory function, although PON-1 activity is impaired by SAA [32]. The absence of an independent association of the HDL anti-inflammatory function as observed here could have been due to the small number of participants. Rather, besides an anticipated effect of PON-1 activity [29, 39–41], an impaired HDL anti-inflammatory capacity was related independently with plasma glucose levels as well as with glycated hemoglobin, thereby probably pointing towards glycation as a potential mechanism. Previous work has established that glycation of apoA-I impairs the anti-inflammatory properties of HDL thereby lending experimental plausibility to such a mechanism [42]. We observed an association of impaired HDL anti-inflammatory function with hs-CRP as global marker of enhanced low-grade inflammation. This relationship expands on previous findings about the impact of inflammation on HDL function [8, 43], although for hs-CRP no direct mechanistic role in affecting HDL’s atheroprotective abilities has been established, yet.

Several methodological considerations and potential limitations of our study need to be appreciated. First, we conducted a cross-sectional study in a relatively small number of participants. As a result, cause-effect relationships cannot be established with certainty. Second, the anti-inflammatory function of HDL was strongly impaired even in T2DM patients with generally acceptable metabolic control. Remarkably, this metric of HDL function was not only associated with fasting plasma glucose but also with the HbA1c level, supporting the notion that chronic hyperglycemic exposure may adversely impact on anti-inflammatory properties of HDL. In this vein, it seems plausible that more severe hyperglycemia could even have a greater impact. Third, we excluded subjects who used lipid lowering treatment. As a result, it is likely that diabetic subjects with mild lipoprotein abnormalities were preferentially included in our study. Accordingly, apoB-containing lipoprotein levels were not elevated in the presently included diabetic subjects. However, this might limit extrapolation of the current findings to subjects with more severe dyslipidemia. Fourth, it would be interesting to assess determinants of HDL subpopulations and HDL particle numbers, measures of HDL that are linked to function and provide clinical information beyond HDL-C mass measurements [10–14]. Lastly, it is important to note that the biological response of HUVECs is variable [44]. Our experimental approach takes this notion into account; all measurements were done on pooled HUVECs from different donors exactly at the same time with identical reagents. Still, these sorts of HDL function assays are not comparable to clinical chemistry determinations.

While we previously showed that impaired anti-inflammatory function of HDL predicts recurrent CVD events in myocardial infarction patients [25], prospective studies seem also warranted to explore the impact of this metric of HDL function on the future development of CVD in T2DM. Moreover, the robust independent association of the anti-inflammatory capacity of HDL with hyperglycemia underscores the need to explore the effect of tight metabolic control on HDL function in future studies.

## Conclusions

Our present results are consistent with the concept that T2DM relatively early in the course of the disease exerts a substantial negative impact on a critical atheroprotective function of HDL, namely protection against endothelial inflammation. Our data indicate that even mild hyperglycemia already significantly decreases this important metric of HDL function.

## Abbreviations

apoA-I: apolipoprotein A-I
apoB: apolipoprotein B
BMI: body mass index
CVD: cardiovascular disease
HDL: high density lipoproteins
HbA1c: glycated haemoglobin
hs-CRP: high sensitivity C-reactive protein
LDL: low density lipoproteins
mRNA: messenger ribonucleic acid
SAA: serum amyloid A
PON-1: paraoxonase-1
T2DM: type 2 diabetes mellitus
TNF-α: tumor necrosis factor-α
VCAM-1: vascular cell adhesion molecule-1

## References

[1] Mazzone T, Chait A, Plutzky J (2008). Cardiovascular disease risk in type 2 diabetes mellitus: insights from mechanistic studies. Lancet.

[2] Lehto S, Rönnemaa T, Haffner SM, Pyörälä K, Kallio V, Laakso M (1997). Dyslipidemia and hyperglycemia predict coronary heart disease events in middle-aged patients with NIDDM. Diabetes.

[3] Lewington S, Whitlock G, Clarke R, Prospective Studies Collaboration (2007). Blood cholesterol and vascularmortality by age, sex, and blood pressure: a meta-analysis of individual data from 61 prospective studies with 55,000 vascular deaths. Lancet.

[4] The Emerging Risk Factors Collaboration (2009). Major lipids, apolipoproteins, and risk of vascular disease. JAMA.

[5] Kappelle PJ, van Tol A, Wolffenbuttel BH, Dullaart RP (2011). Cholesteryl ester transfer protein inhibition in cardiovascular risk management: ongoing trials will end the confusion. Cardiovasc Ther.

[6] Voight BF, Peloso GM, Orho-Melander M (2012). Plasma HDL cholesterol and risk of myocardial infarction: a mendelian randomisation study. Lancet.

[7] Rader DJ (2016). New therapeutic approaches to the treatment of dyslipidemia. Cell Metab.

[8] Triolo M, Annema W, Dullaart RP, Tietge UJ (2013). Assessing the functional properties of high-density lipoproteins: an emerging concept in cardiovascular research. Biomark Med.

[9] Rye KA, Barter PJ (2014). Cardioprotective functions of HDLs. J Lipid Res.

[10] Camont L, Chapman MJ, Kontush A (2011). Biological activities of HDL subpopulations and their relevance to cardiovascular disease. Trends Mol Med.

[11] Mackey RH, Greenland P, Goff DC (2012). High-density lipoprotein cholesterol and particle concentrations, carotid atherosclerosis, and coronary events: MESA (multi-ethnic study of atherosclerosis). J Am Coll Cardiol.

[12] Khera AV, Demler OV, Adelman SJ (2017). Cholesterol efflux capacity, high-density lipoprotein particle number, and incident cardiovascular events: an analysis From the JUPITER trial (justification for the use of statins in prevention: an intervention trial evaluating rosuvastatin). Circulation.

[13] Mascarenhas-Melo F, Marado D, Palavra F (2013). Diabetes abrogates sex differences and aggravates cardiometabolic risk in postmenopausal women. Cardiovasc Diabetol.

[14] Mascarenhas-Melo F, Sereno J, Teixeira-Lemos E (2013). Implication of low HDL-c levels in patients with average LDL-c levels: a focus on oxidized LDL, large HDL subpopulation, and adiponectin. Mediat Inflamm.

[15] de Vries R, Groen AK, Perton FG (2008). Increased cholesterol efflux from cultured fibroblasts to plasma from hypertriglyceridemic type 2 diabetic patients: roles of pre beta-HDL, phospholipid transfer protein and cholesterol esterification. Atherosclerosis.

[16] Machado-Lima A, Iborra RT, Pinto RS (2015). In type 2 diabetes mellitus glycated albumin alters macrophage gene expression impairing ABCA1-mediated cholesterol efflux. J Cell Physiol.

[17] Annema W, Dikkers A, de Boer JF (2016). Impaired HDL cholesterol efflux in metabolic syndrome is unrelated to glucose tolerance status: the CODAM study. Sci Rep.

[18] Kontush A, Chapman MJ (2010). Antiatherogenic function of HDL particle subpopulations: focus on antioxidative activities. Curr Opin Lipidol.

[19] Nobécourt E, Jacqueminet S, Hansel B (2005). Defective antioxidative activity of small dense HDL3 particles in type 2 diabetes: relationship to elevated oxidative stress and hyperglycaemia. Diabetologia.

[20] Gowri MS, Van der Westhuyzen DR, Bridges SR, Anderson JW (1999). Decreased protection by HDL from poorly controlled type 2 diabetic subjects against LDL oxidation may be due to the abnormal composition of HDL. Arterioscler Thromb Vasc Biol.

[21] Morgantini C, Natali A, Boldrini B (2011). Anti-inflammatory and antioxidant properties of HDLs are impaired in type 2 diabetes. Diabetes.

[22] Kappelle PJ, de Boer JF, Perton FG (2012). Increased LCAT activity and hyperglycaemia decrease the antioxidative functionality of HDL. Eur J Clin Investig.

[23] Cockerill GW, Rye KA, Gamble JR (1995). High-density lipoproteins inhibit cytokine-induced expression of endothelial cell adhesion molecules. Arterioscler Thromb Vasc Biol.

[24] Patel PJ, Khera AV, Jafri K (2011). The anti-oxidative capacity of high density lipoprotein is reduced in acute coronary syndrome but not in stable coronary artery disease. J Am Coll Cardiol.

[25] Dullaart RP, Annema W, Tio RA, Tietge UJ (2014). The HDL anti-inflammatory function is impaired in myocardial infarction and may predict new cardiac events independent of HDL cholesterol. Clin Chim Acta.

[26] Annema W, Willemsen HM, de Boer JF (2016). HDL function is impaired in acute myocardial infarction independent of plasma HDL cholesterol levels. J Clin Lipidol.

[27] Grundy SM, Cleeman JI, Daniels SR (2011). Diagnosis and management of the metabolic syndrome: an American Heart Association/National Heart, Lung, and Blood Institute Scientific Statement. Circulation.

[28] Dullaart RP, de Vries R, Sluiter WJ, Voorbij HA (2009). High plasma C-reactive protein (CRP) is related to low paraoxonase-I (PON-I) activity independently of high leptin and low adiponectin in type 2 diabetes mellitus. Clin Endocrinol (Oxf).

[29] Kunutsor SK, Bakker SJ, James RW, Dullaart RP (2016). Serum paraoxonase-1 activity and risk of incident cardiovascular disease: the PREVEND study and meta-analysis of prospective population studies. Atherosclerosis.

[30] van Himbergen TM, Roest M, de Graaf J (2005). Indications that paraoxonase-1 contributes to plasma high density lipoprotein levels in familial hypercholesterolemia. J Lipid Res.

[31] Hazenberg BP, Limburg PC, Bijzet J, van Rijswijk MH (1999). A quantitative method for detecting deposits of amyloid A protein in aspirated fat tissue of patients with arthritis. Ann Rheum Dis.

[32] Kappelle PJ, Bijzet J, Hazenberg BP, Dullaart RP (2011). Lower serum paraoxonase-1 activity is related to higher serum amyloid a levels in metabolic syndrome. Arch Med Res.

[33] Dullaart RP, de Vries R, van Tol A, Sluiter WJ (2007). Lower plasma adiponectin is a marker of increased intima-media thickness associated with type 2 diabetes mellitus and with male gender. Eur J Endocrinol.

[34] Nijstad N, de Boer JF, Lagor WR (2011). Overexpression of apolipoprotein O does not impact on plasma HDL levels or functionality in human apolipoprotein A-I transgenic mice. Biochim Biophys Acta.

[35] Sorrentino SA, Besler C, Rohrer L (2010). Endothelial-vasoprotective effects of high-density lipoprotein are impaired in patients with type 2 diabetes mellitus but are improved after extended-release niacin therapy. Circulation.

[36] Griffiths K, Pazderska A, Ahmed M (2017). Type 2 diabetes in young females results in increased serum amyloid A and changes to features of high density lipoproteins in both HDL2 and HDL3. J Diabetes Res.

[37] Tölle M, Huang T, Schuchardt M (2012). High-density lipoprotein loses its anti-inflammatory capacity by accumulation of pro-inflammatory-serum amyloid A. Cardiovasc Res.

[38] Tietge UJ, Maugeais C, Lund-Katz S (2002). Human secretory phospholipase A2 mediates decreased plasma levels of HDL cholesterol and apoA-I in response to inflammation in human apoA-I transgenic mice. Arterioscler Thromb Vasc Biol.

[39] Murakami H, Tanabe J, Tamasawa N (2013). Reduction of paraoxonase-1 activity may contribute the qualitative impairment of HDL particles in patients with type 2 diabetes. Diabetes Res Clin Pract.

[40] Shen Y, Ding FH, Sun JT (2015). Association of elevated apoA-I glycation and reduced HDL-associated paraoxonase1, 3 activity, and their interaction with angiographic severity of coronary artery disease in patients with type 2 diabetes mellitus. Cardiovasc Diabetol.

[41] Patra SK, Singh K, Singh R (2013). Paraoxonase 1: a better atherosclerotic risk predictor than HDL in type 2 diabetes mellitus. Diabetes Metab Syndr.

[42] Nobécourt E, Tabet F, Lambert G (2010). Nonenzymatic glycation impairs the antiinflammatory properties of apolipoprotein A-I. Arterioscler Thromb Vasc Biol.

[43] Feingold KR, Grunfeld C (2016). Effect of inflammation on HDL structure and function. Curr Opin Lipidol.

[44] Méndez-Cruz AR, Paez A, Jiménez-Flores R (2007). Increased expression of inflammation-related co-stimulatory molecules by HUVECs from newborns with a strong family history of myocardial infarction stimulated with TNF-alpha and oxLDL. Immunol Lett.

